# Efficient and synergistic removal of tetracycline and Cu(II) using novel magnetic multi-amine resins

**DOI:** 10.1038/s41598-018-23205-9

**Published:** 2018-03-19

**Authors:** Zengyin Zhu, Mancheng Zhang, Wei Wang, Qing Zhou, Fuqiang Liu

**Affiliations:** 1 State Key Laboratory of Pollution Control and Resources Reuse, School of the Environment, Nanjing University, Nanjing, 210023 PR China; 2 Jiangsu Province Key Laboratory of Environmental Engineering, Jiangsu Provincial Academy of Environmental Science, Nanjing, 210036 PR China

## Abstract

A series of magnetic multi-amine resins (MMARs, named E1D9-E9D1) was proposed for the removal of tetracycline (TC) and Cu(II) in sole and binary solutions. Results showed that the N content of the resins increased sharply from 1.7% to 15.49%, and the BET surface areas decreased from 1433.4 m^2^/g to 8.9 m^2^/g with methyl acrylate ratio increasing from E1D9 to E9D1. Their adsorption capacities for TC and Cu(II) could reach 0.243 and 0.453 mmol/g, respectively. The adsorption isotherms of TC onto MMARs transformed from heterogeneous adsorption to monolayer-type adsorption with DVB monomer ratio in resin matrix decrease, suggesting the dominant physical adsorption between TC and benzene rings. TC adsorption capacity onto E9D1 was higher than that onto E7D3 when the equilibrium concentration of TC exceeded 0.043 mmol/L because the electrostatic interaction between negatively charged groups of TC and protonated amines of adsorbents could compensate for the capacity loss resulting from BET surface area decrease. In the binary system, the electrostatic interaction between negatively charged TC-Cu(II) complex and protonated amines of adsorbents was responsible for the synergistic adsorption onto E7D3 and E9D1. The XPS spectra of magnetic resins before and after adsorption were characterized to prove the probable adsorption mechanisms. This work provides alternative adsorbent for the efficient treatment of multiple pollution with different concentrations of organic micropollutants and heavy metal ions.

## Introduction

Organic and inorganic pollutants, such as organic micropollutants (OMPs) and heavy metals, widely exist in environment, resulting in multiple pollution. Such pollutants were discharged into the environment with breeding wastewater, electroplating effluent, and municipal sewage^1^. The multiple pollutants in aquatic water have significantly affected the ecosystem security due to their combined toxicity and carcinogenicity. The combination of oxytetracycline and Cu(II) showed greater suppression effect for Shannon’s diversity and evenness of microbial community compared to individual one of the contaminants^2^. The complex of oxytetracycline and ciprofloxacin with copper, zinc, and cadmium exhibited the highest toxic and predominantly correlated with toxicity to their mixture^3^. The coexistence of pentachlorophenol and copper enhanced cytotoxic influence in a synergistic mode^4^. Consequently, the effective removal of the multiple pollutants of OMPs and heavy metal has gained a growing attention.

The methods of OMPs and copper removal involving chemical precipitation, advanced oxidation, membrane treatment, adsorption, and biological treatment^5–10^. Therein, adsorption, a relatively simple and recyclable method, has been widely applied for OMPs and copper removal. Various adsorbents, such as activated carbon, minerals, chitosan, and polymers, were utilized for the removal of multiple pollutant^11–15^. Among them, synthetic polymers trigger numerous studies due to their excellent stability, versatile structure, and easy regeneration.

Previous studies have proved that copper can be adsorbed through the chelation with amine groups of resin^16,17^, and hypercrosslinked resin possesses excellent performance for OMP adsorption through physical interaction due to its great surface area^18^. Ling’s work had proven that chelating resin could synergistically remove tetracycline and copper, and tetracycline adsorption was enhanced approximately 13 times in the presence of copper^19^. However, the chelating resin had poor adsorption performance for tetracycline without or with low initial concentration of copper. Besides, the conventional non-magnetic resin had to be used in a fixed-bed column, which limited the flux due to the high resistance. Therefore, exploring adsorbents for conveniently synergistic removal of OMPs, heavy metal ions, and their multiple pollutants is important.

In this work, a series of magnetic multi-amines decorated resins (MMARs) with different monomer ratios were developed for the removal of multiple contaminants. The tetracycline (TC, an antibiotic) and copper both existing in breeding wastewater and municipal sewage^13^, were selected as the target compounds. The adsorption behavior of TC and Cu(II) onto MMARs in sole and binary solution was investigated. The roles of monomers and interaction processes were revealed through the adsorption behavior in sole and binary systems as well as in XPS characterization.

## Materials and Methods

### Materials

Divinylbenzene (DVB, 80 wt.%), methylacrylate (MA, >99%), benzoyl peroxide, and TC were obtained from J&K Chemical Co., Ltd., China. Tetraethylenepentamine, anhydrous ferric chloride, 1,2-dichloroethane, sodium chloride, hydrochloric acid, sodium hydroxide, and cupric nitrate trihydrate were all analytical reagents and purchased from Sinopharm Chemical Reagent Co., Ltd (China). DVB and MA were passed through a strong base anion exchange resin column to remove the inhibitors prior to use.

### Preparation of MMARs

MMARs were prepared through copolymerization, post-crosslinking, and amination. The magnetic nanoparticles were obtained through chemical precipitation and surface modification, which was described elsewhere^20^. For the copolymerization process, the oil phase containing DVB, MA, toluene, surface-modified magnetic nanoparticles, and benzoyl peroxide was introduced into a three-necked flask, followed by the addition of the aquatic phase consisting of sodium chloride, water, and gelatin as the dispersant. The mixture was heated to 353 K maintaining for 12 h at 400 rpm. The obtained polymer beads were rinsed with deionized water and ethanol three times for each, and then the post-crosslinking process was conducted as reported in previous work^21,22^. Finally, the magnetic beads were mixed with tetraethylenepentamine and heated at 393 K for 20 h. The resultant MMAR was rinsed to neutral and dried at 333 K. A series of MMARs was obtained with different monomer ratios through the above described procedure.

### Adsorption assay

Batch experiments were conducted to investigate the adsorption behavior of TC and Cu(II) onto MMARs. As to the adsorption kinetics, 0.01 g of MMAR was introduced into 100 mL of TC or Cu(II) solution. The initial TC concentration was 0.05 mmol/L, and the Cu(II) concentration was 0.1 mmol/L. The adsorption system was performed at 293 K and sampled at diverse time intervals. For the adsorption isotherms, 0.01 g of MMAR was added into a series of 150 mL conical flasks containing 100 mL of TC or Cu(II) solution with different initial concentrations.

For the binary adsorption, 0.01 g MMAR was added into a series of 150 mL conical flasks with 100 mL of TC solutions (0.05 mmol/L). Different amounts of cupric nitrate trihydrate were added into the above solutions to obtain Cu(II) initial concentrations of 0.05, 0.1, 0.2, and 0.5 mmol/L. To investigate the adsorption behavior of Cu(II) in the presence of TC, the binary solutions containing Cu(II) (0.1 mmol/L) and TC (0.025, 0.05, and 0.1 mmol/L) were shaken with 0.01 g MMAR.

The solution pH was adjusted by 0.1 mol/L HCl or NaOH solution to investigate the influence of pH.

The concentration of Cu(II) was determined by atomic absorption spectrophotometer (Thermo ICE3500, USA). The limit of detection (LOD), limit of quantification (LOQ), and relative standard deviation (RSD) of this method were 0.23 μmol/L, 0.78 μmol/L and 0.06, respectively, which were listed in Table S1. The TC concentration was analyzed by UV–visible spectrometry (SHIMADZU, UV-1800, Japan) at 360 nm. The LOD, LOQ and RSD of UV-Vis were 0.305 μmol/L, 1.01 μmol/L and 0.62, respectively. The sample pH was adjusted to 1–2 to decompose the TC-Cu(II) complex in solution prior to analysis. All assays were conducted three times and the average data were adopted.

### Characterization

The magnetization curves were measured by a vibrating sample magnetometer (VSM, Quantum Design MPMS-5S, USA). The dried MMARs particles were coated by gold (40–50 nm) and then observed by a scanning electron microscope (SEM, S-3400N II, Hitachi,Japan) with an accelerating voltage of 20 kV. The specific surface area and pore structure were calculated using BET and BJH equations, respectively, with the N_2_ adsorption–desorption data, and calculation was performed automatically on ASAP 2010 (Micromeritics, USA). The fresh resin and saturated resin were characterized with X-ray photoelectron spectroscopy (PHI 5000 VersaProbe, UlVAC-PHI Japan). The obtained spectra were analyzed by XPSPEAK41 software and calibrated against the neutral C1s band at 284.6 eV to eliminate the surface charging effects and systematic errors.

## Results and Discussion

### Adsorbents preparation

Previous studies have proven that acrylic chelating resin and hypercrosslinked resin show great removal performance for heavy metal ions and organic micropollutants, respectively^18,23–25^. In an effort to synthesize novel adsorbent for simultaneous removal of heavy metal ions and organic micropollutants, the preparation methods of acrylic chelating resin and hypercrosslinked resin were combined. DVB was employed as monomer to provide crosslinking network and adsorption sites for organic micropollutants. Methyl acrylate (MA) was used as the ester monomer to provide reactive sites for multi-amines group. The preparation procedures of magnetic multi-amines resins (MMARs) with different ester monomers are shown in Figure S1.

### Characterization

**Figure 1:**
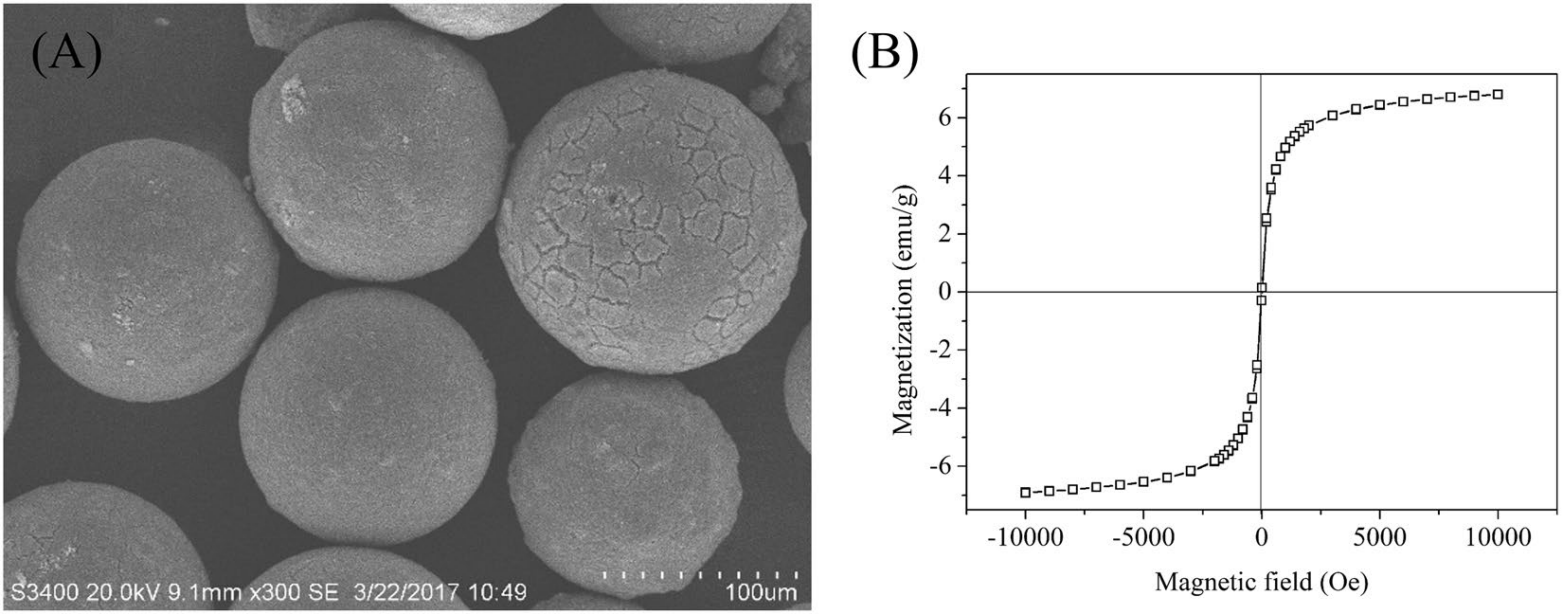
SEM image (**A**) and magnetization curves (**B**) of E1D9.

As all MMARs had similar surface morphology and magnetic property, the SEM image and magnetization curves of E1D9 were exhibited in Fig. 1 as representative. The diameters of MMARs ranged from 100 µm to 150 µm, and they possessed rough surface due to the presence of magnetic nanoparticles. All MMARs had similar specific saturation magnetization, which was attributed to the same dosage of magnetic nanoparticles in the preparation processes. The magnetization curve of MMARs is depicted in Fig. 1B. MMARs had a specific saturation magnetization of 6.9 emu/g, which was suitable for the application in a completely mixed contactor and could be separated from solution by an ordinary magnet^26^.

The physicochemical properties of a series of magnetic multi-amine-decorated resins are listed in Table 1. The BET surface areas and pore volumes of magnetic resins increased sharply with increasing DVB ratio. The surface area of E1D9 was approximately 161 times larger than that of E9D1. Greater linear positive correlation between BET surface areas and DVB ratio was observed (Figure S2). These results demonstrated that resin matrix and channel network were mainly supported by DVB. The N content of E1D9 was 1.7% and that of E9D1 was 15.49%. A positive correlation exists between N content and MA ratio in matrix.

**Table 1:** Physicochemical properties of magnetic multi-amines decorated resins.

		E1D9	E3D7	E5D5	E7D3	E9D1
Ester monomer: DVB (m/m)		10:90	30:70	50:50	70:30	90:10
BET surface area (m^2^/g)		1433.4	922.7	612.5	193.8	8.9
Micropore area (m^2^/g)		417	350	186.2	51.0	3.0
Pore volume (cm^3^/g)		2.814	1.51	1.18	0.49	0.01
Average pore diameter (nm)		6.912	5.998	6.07	4.008	4.116
	C (%)	84.13	72.55	63.38	52.66	41.21
Elemental analysis	H (%)	7.54	7.21	7.41	7.65	7.65
	N (%)	1.7	4.53	7.24	10.77	15.49

### Adsorption kinetics in sole solute

**Figure 2:**
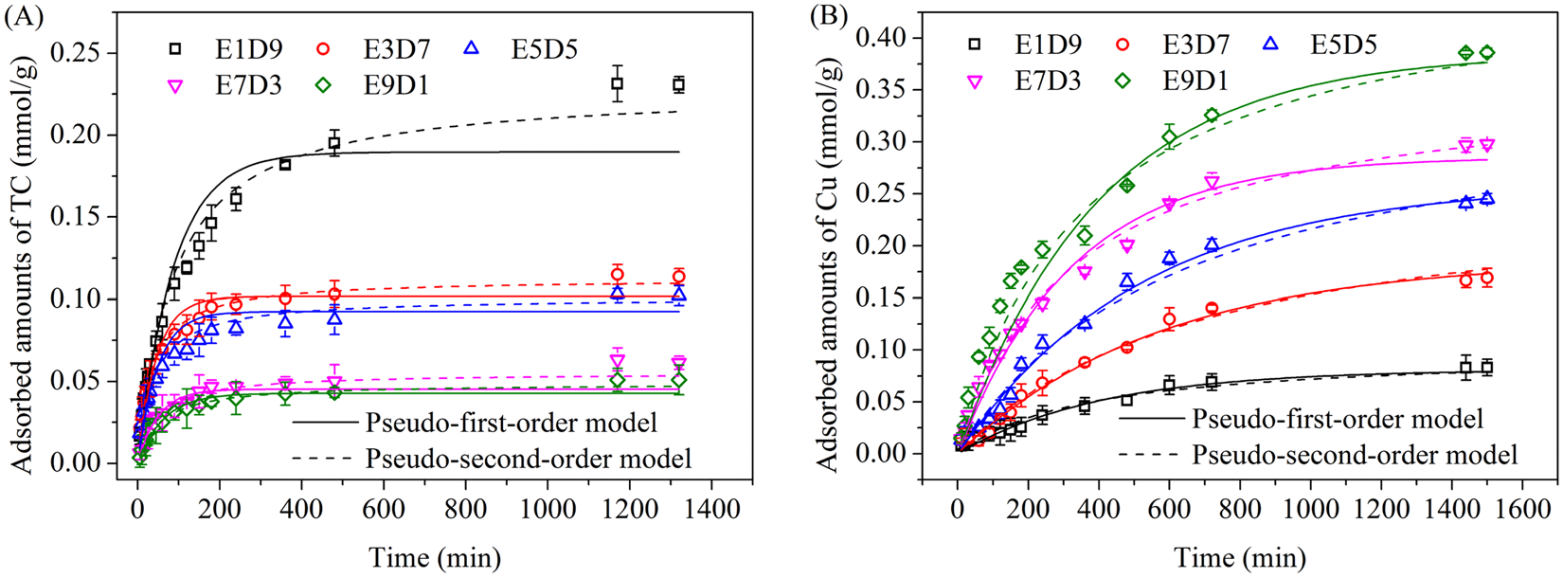
Adsorption kinetics of (**A**) TC and (**B**) Cu(II) onto MMARs.

DVB monomer in resin matrix supported the resin frame and provided the isolated double bonds, which made it possible to perform the post-crosslinking reaction and increase the specific surface area of resin. Meanwhile, the DVB monomer is more hydrophobic than methyl acrylate monomer. Thus, higher DVB ratio in resin matrix means higher hydrophobicity of the resin. The previous works revealed that tetracycline molecules were adsorbed on hypercrosslinked resin with the sole DVB monomer mainly through hydrophobic interaction and π–π interaction, and higher specific surface area resulted in higher adsorbed amount^18^. The adsorption kinetics of TC onto MMARs at 283 K is shown in Fig. 2. The adsorption capacities of TC onto MMARs increased with increasing DVB ratio in resin matrix. These results proved that the adsorption of TC might be mainly through hydrophobic interaction and π–π interaction.

As shown in Fig. 2, the adsorption capacities of Cu(II) onto MMARs increased significantly from E1D9 to E9D1. It was due to the higher ester monomer ratio in resin matrix providing more grafting sites for multi-amine groups, which could capture Cu(II) through chelation.

Pseudo-first-order and pseudo-second-order models were utilized to fit the kinetic data. The fitting parameters are listed in Table 2. The adsorption of TC on all MMARs fitted the pseudo-second-order models better with larger correlation coefficient (R^2^). The equilibrium absorption capacity (*q*_*e*_ value) decreased with decreasing DVB ratio and surface area. The *k*_2_ (rate constants) values for TC adsorption onto MMARs increased from E1D9 to E9D1, suggesting the faster adsorption kinetics of MMARs with lower DVB ratio.

**Table 2:** Kinetic parameters for TC and Cu(II) adsorption onto MMARs.

		Pseudo-first-order model			Pseudo-second-order model		
		*k*_1_(10^−2^)	*Q*_*e*_	R^2^	*k*_2_(10^−2^)	*q*_*e*_	R^2^
TC	E1D9	0.75	0.213	0.9597	3.76	0.243	0.9906
	E3D7	2.22	0.101	0.9428	25.38	0.112	0.9893
	E5D5	2.09	0.088	0.9004	26.63	0.098	0.9742
	E7D3	1.49	0.053	0.9282	30.16	0.060	0.9771
	E9D1	1.52	0.044	0.9269	37.14	0.050	0.9890
Cu	E1D9	0.22	0.086	0.9792	1.82	0.112	0.9808
	E3D7	0.19	0.181	0.9856	0.63	0.248	0.9814
	E5D5	0.20	0.259	0.9899	0.48	0.351	0.9853
	E7D3	0.29	0.295	0.9842	0.80	0.366	0.9926
	E9D1	0.32	0.372	0.9637	0.74	0.453	0.9839

Cu(II) adsorption onto MMARs fitted both pseudo-first-order and pseudo-second-order well with correlation coefficient (R^2^) larger than 0.96. The equilibrium adsorption capacity (*Q*_*e*_ and *q*_*e*_ values) increased with MA ratio increasing in resin matrix. More MA ratio in the matrix indicates more grafting sites for multi-amine groups and higher N content of MMAR that can capture more Cu(II) from solution.

### Adsorption isotherms in sole solute

**Figure 3:**
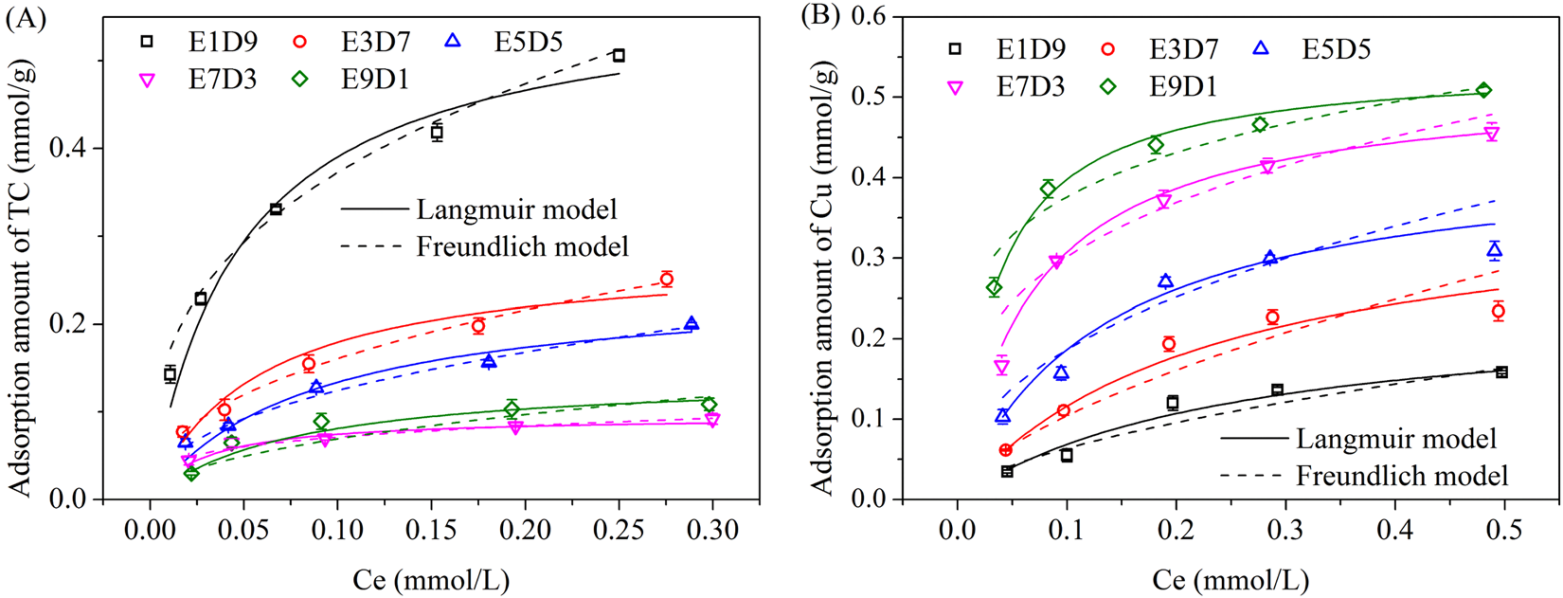
Adsorption isotherms of (**A**) TC and (**B**) Cu(II) onto MMARs.

The adsorption isotherms of TC and Cu(II) on MMARs at 293 K are described in Fig. 3. Langmuir and Freundlich models were used to fit the isotherm data, and the fitting parameters are listed in Table 3. The adsorption of Cu(II) onto MMARs was described better by Langmuir model. The *Q*_*m*_ (maximum adsorbed amount) and *K*_*L*_ (adsorption equilibrium constant) values increased with increasing ester monomer ratio. Larger N content indicates more chelation sites and more easily capturing Cu(II).

**Table 3:** Adsorption isotherm parameters of TC and Cu(II) adsorption onto MMARs.

		Langmuir			Freundlich		
		*Q*_*m*_	*k*_*L*_	R^2^	*K*_*F*_	*n*	R^2^
TC	E1D9	0.55	25.25	0.9654	0.85	2.71	0.9898
	E3D7	0.30	13.76	0.9448	0.43	2.31	0.9929
	E5D5	0.23	14.67	0.9383	0.33	2.42	0.9879
	E7D3	0.09	42.29	0.9353	0.12	4.24	0.9405
	E9D1	0.13	19.54	0.9521	0.18	2.82	0.8297
Cu	E1D9	0.25	3.72	0.9518	0.25	1.78	0.8963
	E3D7	0.33	6.07	0.9485	0.36	2.16	0.8518
	E5D5	0.41	8.29	0.9439	0.45	2.49	0.8455
	E7D3	0.53	12.52	0.9916	0.61	2.98	0.9082
	E9D1	0.53	29.40	0.9879	0.61	4.62	0.9172

The adsorption of TC onto MMARs with high DVB monomer ratio was described better by Freundlich model with larger correlation coefficient (R^2^). However, the adsorption behavior of TC onto E9D1 fitted Langmuir model better, which indicates that the adsorption of TC onto MMARs transformed from heterogeneous adsorption to monolayer-type adsorption with the DVB monomer ratio in resin matrix decreases from 90% to 10%. The heterogeneous adsorption of MMAR with high DVB monomer ratio agreed with the adsorption behavior of TC onto DVB-matrical magnetic hypercrosslinked resin^27^. The dominant heterogeneous adsorption of TC onto MMARs with high DVB monomer ratio (>30%) further proved that DVB matrix was in favor of TC adsorption. TC adsorption onto E9D1 was described better by Langmuir model indicating that some other adsorption mechanisms, except physical interaction, occurred in TC adsorption onto E9D1.

As shown in Fig. 3A, TC adsorption on E7D3 was higher than that on E9D1 with an equilibrium concentration of TC lower than 0.043 mmol/L due to the higher surface area of E7D3, which was in accordance with the adsorption behavior in kinetics study. However, the adsorbed amounts of TC on E7D3 was lower than that on E9D1 when the equilibrium concentration of TC exceeded 0.043 mmol/L. Lower surface area of E9D1 indicated less physical adsorption sites for TC, whereas higher N content of E9D1 provided more protonated amines, which can react with negatively charged tricarbonyl group of TC through electrostatic interaction. The adsorbed amounts through electrostatic interaction could make up for the capacity loss resulting from decreasing BET surface area when TC molecules and protonated amine groups on resin were both sufficient.

### Effect of solution pH in sole solute

Considering the precipitation limits of Cu(II) and the stability limits of magnetic resins, the effect of solution pH ranging from 2–5 on Cu(II) adsorption was evaluated, and the solution pH was adjusted from 2 to 12 for TC adsorption.

**Figure 4:**
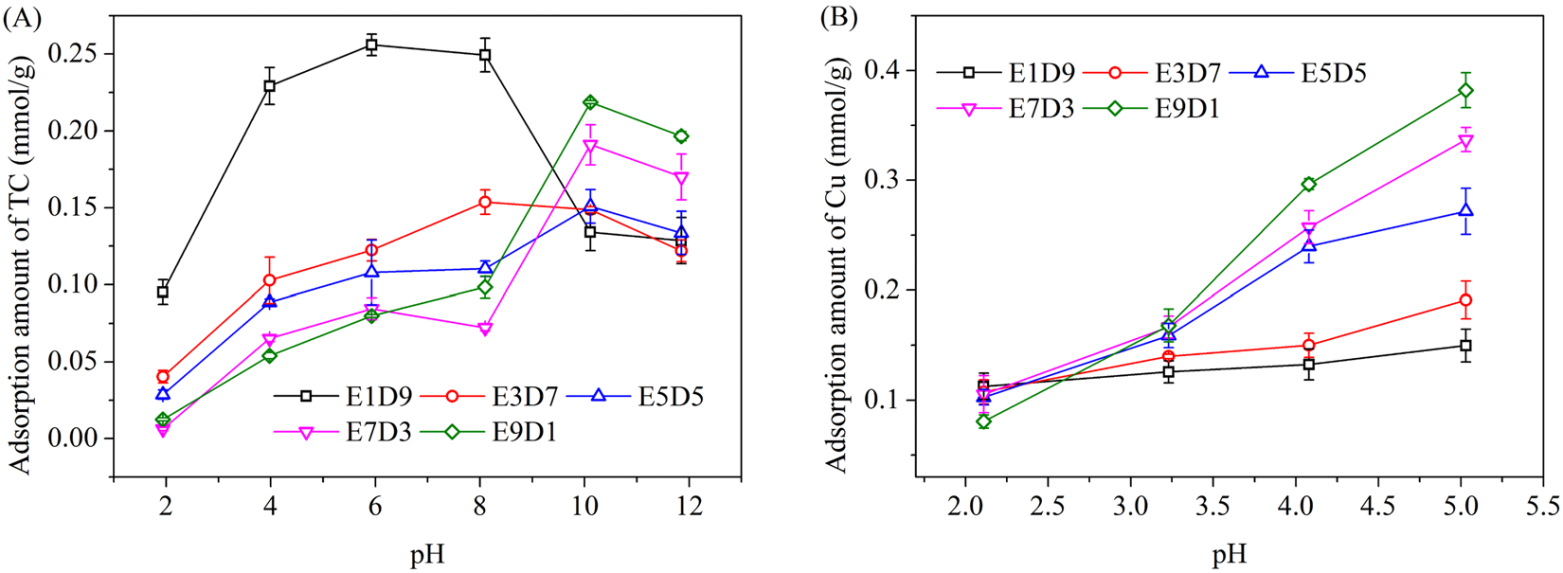
Effect of solution pH on the adsorption of (**A**) TC and (**B**) Cu(II) onto MMARs.

As depicted in Fig. 4A, the adsorption capacity of TC on E1D9 and E3D7 increased with increasing solution pH from 2 to 8 and decreased at solution pH > 8, which was identical to the adsorption behavior of TC onto hypercrosslinked resin^27^. It further confirmed that TC was adsorbed onto E1D9 and E3D7 mainly through the physical interaction between TC molecules and DVB benzene rings, such as hydrophobic interaction and π–π interaction^18^.

The adsorbed amounts of TC onto MMARs with high ester monomer ratio (E5D5–E9D1) increased when solution pH increased from 2 to 10 and then decreased at pH 12. This result suggested that TC molecules could interact with amine groups besides benzene rings. As shown in Figure S3, the point of zero charges of E5D5-E9D1 were all approximately 10. The dominant TC species were mainly negatively charged molecules at pH > 7.68 (pKa), which was plotted in Figure S4. The electrostatic interaction between protonated amines of MMARs and negatively charged TC molecules facilitated TC adsorption at pH ≤ 10. Thus, the adsorbed amounts of TC at pH 10 was in the order E9D1 > E7D3 > E5D5 ≈ E3D7 > E1D9, which was opposite to the order at pH < 8. This result proved the existence of the electrostatic interaction. However, the amine groups gradually became to be deprotonated at pH > 10. The electrostatic interaction decreased progressively, and the adsorption capacities of E5D5-E9D1 mainly dominated by non-electrostatic interaction were on a decline.

As shown in Fig. 4B, the adsorption capacities of Cu(II) onto MMARs increased generally with increasing solution pH. Since the copper molecules exist mainly as the divalent positive ions Cu(II), the protonated amine groups at low solution pH would reject Cu(II) through electrostatic repulsion resulting in low adsorption capacity. The chelation interaction between amine groups and Cu(II) become more effective with solution pH increase resulting in the rise of adsorption capacity.

### Binary adsorption

**Figure 5:**
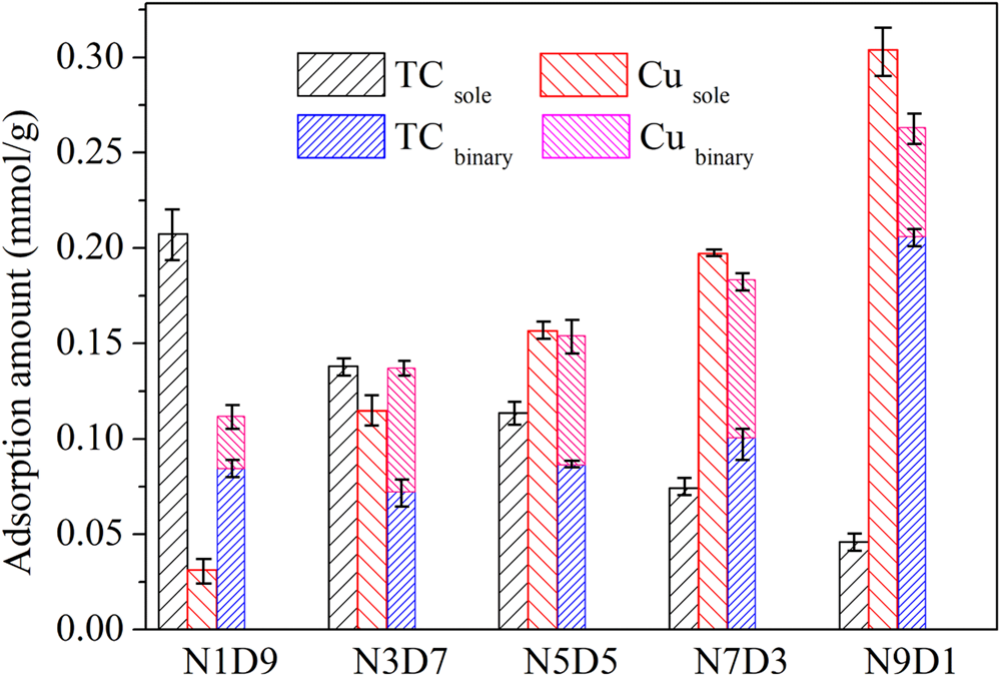
Binary adsorption of TC and Cu(II) onto MMARs.

The adsorption of TC and Cu(II) onto MMARs in binary system is shown in Fig. 5. Compared with the sole system, the adsorption of TC onto MMARs with low ester monomer ratio decreased significantly in the presence of Cu(II). However, TC adsorption onto E7D3 and E9D1 was enhanced by Cu(II) in binary system. This result would be ascribed to the formation of tertiary complexes resin-Cu(II)-TC^28^. For MMARs with low ester monomer ratio, small N content resulted in inability of tertiary complexes to compensate for the capacity loss of physical adsorption.

The adsorption of Cu(II) onto MMARs was all inhibited by TC in binary system. The formation of TC-Cu(II) complex in solution would change the surface charge and molecular size of Cu(II). Meanwhile, TC would inhibit Cu(II) adsorption through blocking channel, competing, and screening interaction sites.

**Figure 6:**
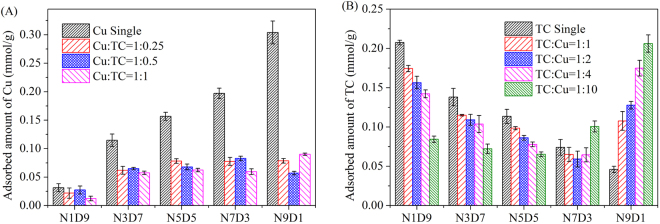
Adsorption of (**A**) Cu(II) and (**B**) TC in binary systems with different initial concentrations.

Due to the specific co-adsorption behavior in binary system, the co-adsorption of TC and Cu(II) with different initial concentrations was investigated and the results are depicted in Fig. 6. The adsorption of Cu onto all MMARs was hindered with the proportion of Cu(II)/TC ranging from 1:0.25 to 1:1, which was observed in Fig. 6A. Similarly, the adsorption of TC onto E1D9-E5D5 were all impeded with the proportion of TC and Cu(II) ranging from 1:1 to 1:10. However, the TC adsorption on E9D1 was enhanced across the TC/Cu(II) range. The TC adsorption onto E7D3 showed different trends with diverse initial concentrations of Cu(II). It was suppression with TC/Cu(II) ranging from 1 to 2, whereas enhancement with TC/Cu(II) ranging from 4 to 10. The adsorbed amount of TC was higher than that in sole system, especially for TC/Cu(II) = 10. It suggested that both suppression and synergy simultaneously occur in TC adsorption in binary system. The synergy would exceed the suppression when both amine groups on MMARs and Cu(II) molecules in solution were abundant.

### XPS characterization

**Figure 7:**
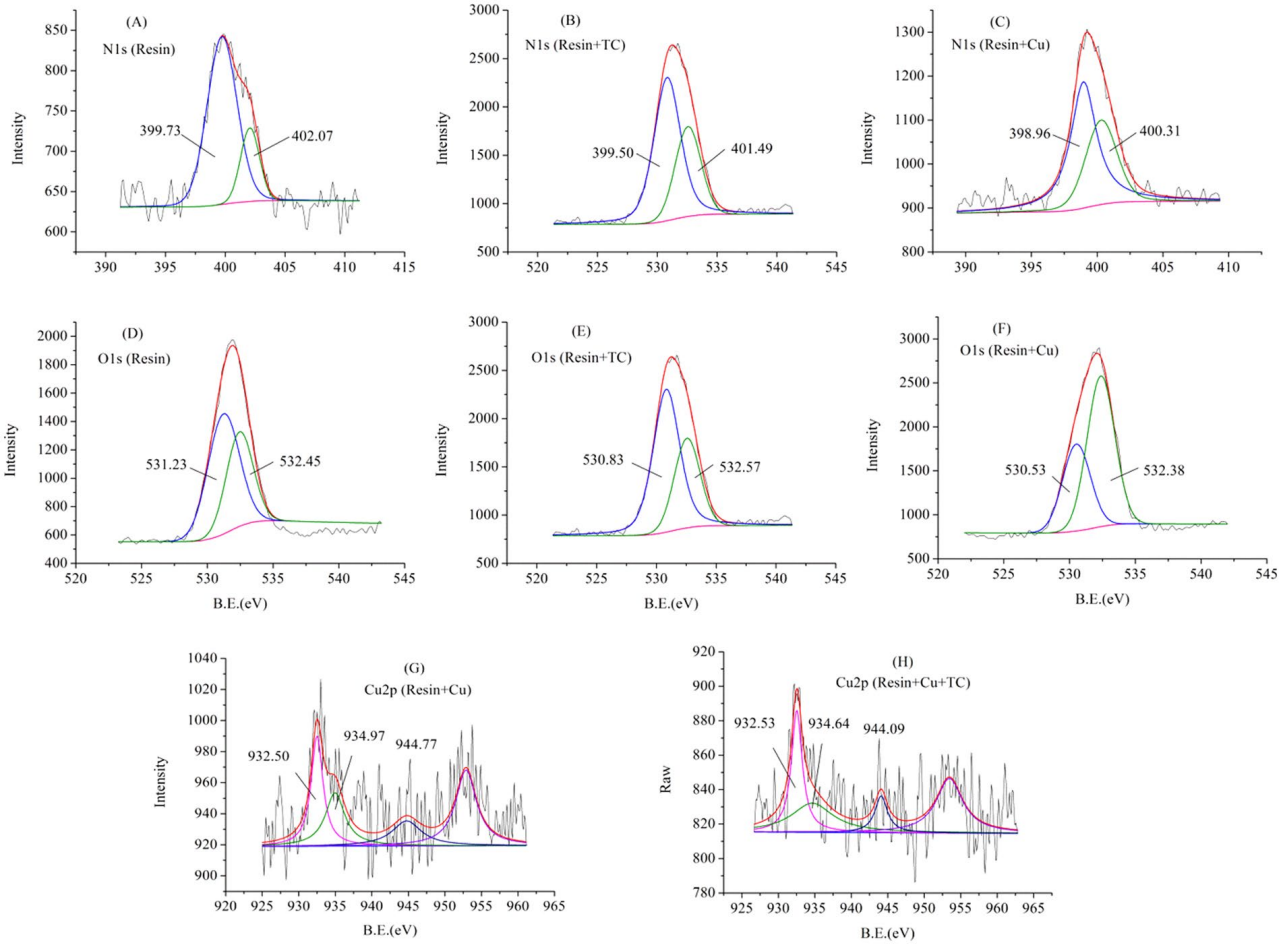
XPS spectra for MMARs before and after adsorption.

In an effort to demonstrate the interaction of copper and TC on the interface of MMARs, XPS spectra were obtained for the fresh and saturated adsorbents. The XPS spectra of fresh resin, Resin + TC, Resin + Cu(II), and Resin + TC + Cu(II) are depicted in Fig. 7.

The N1s spectra of fresh resin were deconvoluted into two peaks at 399.73 and 401.87 eV, which were assigned to the nitrogen in the neutral amines (-NH_2_ or –NH-) and protonated amines (-NH_3_^+^ or –NH_2_^+^-), respectively^14,29,30^. For Resin + Cu(II), both of the peaks for neutral and protonated amines had shifted, suggesting that both of these two kinds of amines participating in the adsorption of copper. The binding energy of the protonated amines decreased by 0.58 eV, and the binding energy of the neutral amines hardly changed, demonstrating that TC reacted with protonated amines.

For the O1s spectra in Fig. 7, the characteristic peak of carbonyl at 531.23 eV shifted to 530.83 and 530.53 eV after adsorbing TC and copper, respectively, indicating that the carbonyl on the resins had a role in both of TC and copper adsorption.

Compared with the XPS spectra of Resin + TC and Resin + Cu(II), the peaks of neutral amines, protonated amines, carbonyl, and hydroxyl for Resin + TC + Cu(II) had shifted. It suggested that all these groups participated in the adsorption in binary system. The binding energy of Cu2p_3/2_ shifted about 0.33 eV due to the formation of TC-Cu(II) complex in binary system.

### Adsorption mechanism

The interaction sites on the MMARs can be classified as the benzene-matrix (Site I), carbonyl (Site II), secondary amine (Site III), and primary amine (Site IV). The hydrophobic and π-π interactions between TC and MMARs almost all react at Site I. TC can be adsorbed at Site II through the H-bonding, and the negatively charged groups of TC can be captured by protonated amines (Sites III and IV) through electrostatic interaction. The electrostatic interaction enhances with increasing ester monomer ratio of MMARs. This result should be responsible for the higher adsorption capacities of E9D1 than that of E7D3. Cu(II) is adsorbed primarily through chelation at Sites II, III, and IV.

In binary system, TC can coordinate with Cu(II) through the amino nitrogen, carbonyl and hydroxyl oxygen atoms, and the negatively charged TC-Cu(II) complex can react with the protonated amines of MMARs through electrostatic interaction, which should account for the promotion of TC adsorption onto E7D3 and E9D1 in the presence of Cu(II).

### Reusability

**Figure 8:**
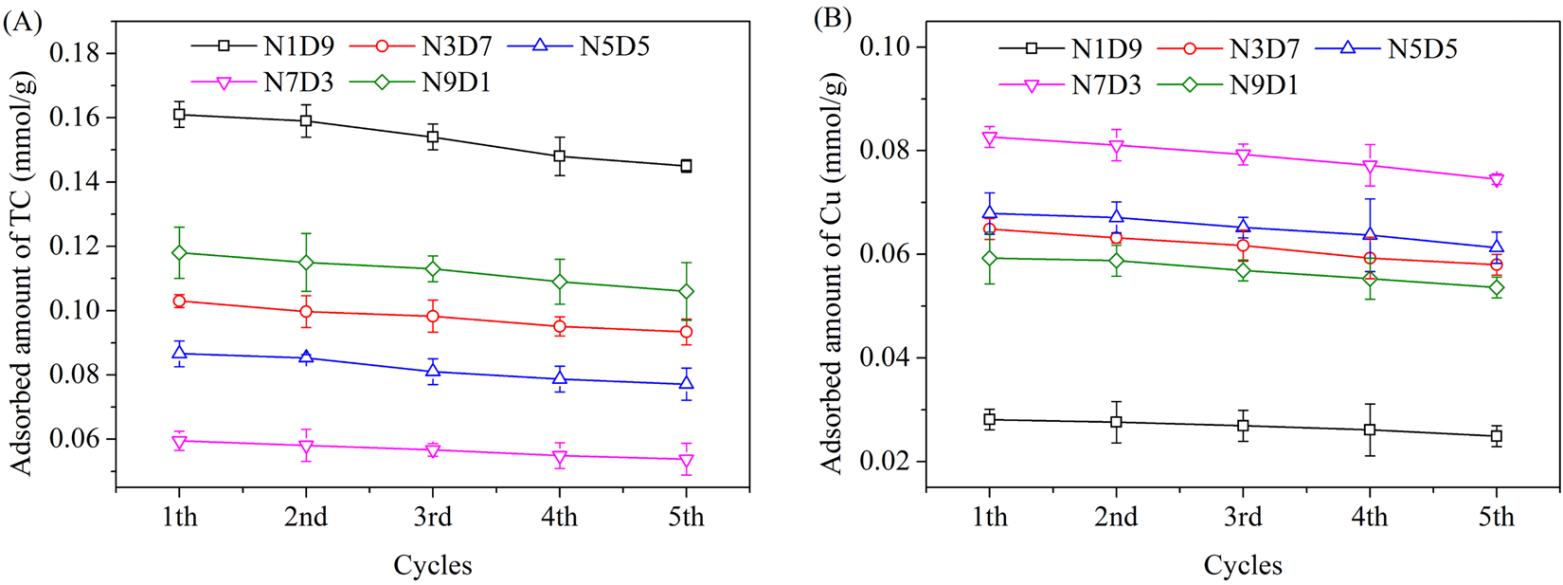
Reusability of MMARs on the adsorption of (**A**) TC and (**B**) Cu(II).

As shown in Fig. 4, the adsorption of TC and Cu(II) were both inhibited at pH 2. Thus, the regeneration of MMAR was carried out with 50 mL of acidized methanol (methanol: 0.01 mol/L HCl = 1: 3, v/v). The acid was used to change the species of target compounds and decrease the affinity between the adsorbates and adsorbents. And then the adsorbates were stripped from adsorbents. Methanol was used to facilitate the release of TC. The adsorption experiments in TC-Cu(II) binary system on MMARs were examined during 5 adsorption-desorption cycles. The results were depicted in Fig. 8. The adsorbed amounts of TC and Cu(II) on MMARs all decreased by about 10% after 5 adsorption-desorption cycles, indicating the good reusability of MMARs.

## Conclusions

A series of magnetic multi-amines resins for the co-adsorption of multiple pollutants were prepared. The adsorption of OMPs and heavy metal ions were mainly through physical adsorption, chelation, and electrostatic interaction. TC was adsorbed onto MMARs through hydrophobic and π-π interactions. The electrostatic interaction between negatively charged groups of TC and protonated amines of MMARs also played a role in TC adsorption, which accounted for the higher adsorption capacities of E9D1 than that of E7D3. Cu(II) adsorption was dominated by chelation. In addition, TC-Cu(II) complex in binary solution could react with protonated amines of MMARs through electrostatic interaction, which was responsible for the enhanced adsorption of TC onto E7D3 and E9D1 in the presence of Cu(II). In practical application, the proper adsorbent could be selected according to the composition of multiple pollutants.

## Electronic supplementary material

Supplementary Materials
