# Supplementary material for: Efficient and synergistic removal of tetracycline and Cu(II) using novel magnetic multi-amine resins
Source: Sci Rep. 2018 Mar 19;8:4762. doi: 10.1038/s41598-018-23205-9 (PMC5859091; doi:10.1038/s41598-018-23205-9)
Supplement: Supplementary file 1 — Supplementary Materials [file 41598_2018_23205_MOESM1_ESM.docx]

Supplementary Materials for

Efficient and synergistic removal of tetracycline and Cu(II) using novel magnetic multi-amine resins

Zengyin Zhu ^a, b^, Mancheng Zhang ^b^, Wei Wang ^b^, Qing Zhou ^a^, Fuqiang Liu ^a,^ ^*^

^a^ State Key Laboratory of Pollution Control and Resources Reuse, School of the Environment, Nanjing University, Nanjing 210023, PR China

^b^ Jiangsu Province Key Laboratory of Environmental Engineering, Jiangsu Provincial Academy of Environmental Science, Nanjing 210036, PR China

*Corresponding Author

Tel.: +86 139 1387 1032; fax: +86 25 89680377.

E-mail: [jogia@163.com](mailto:jogia@163.com) (F. Liu).


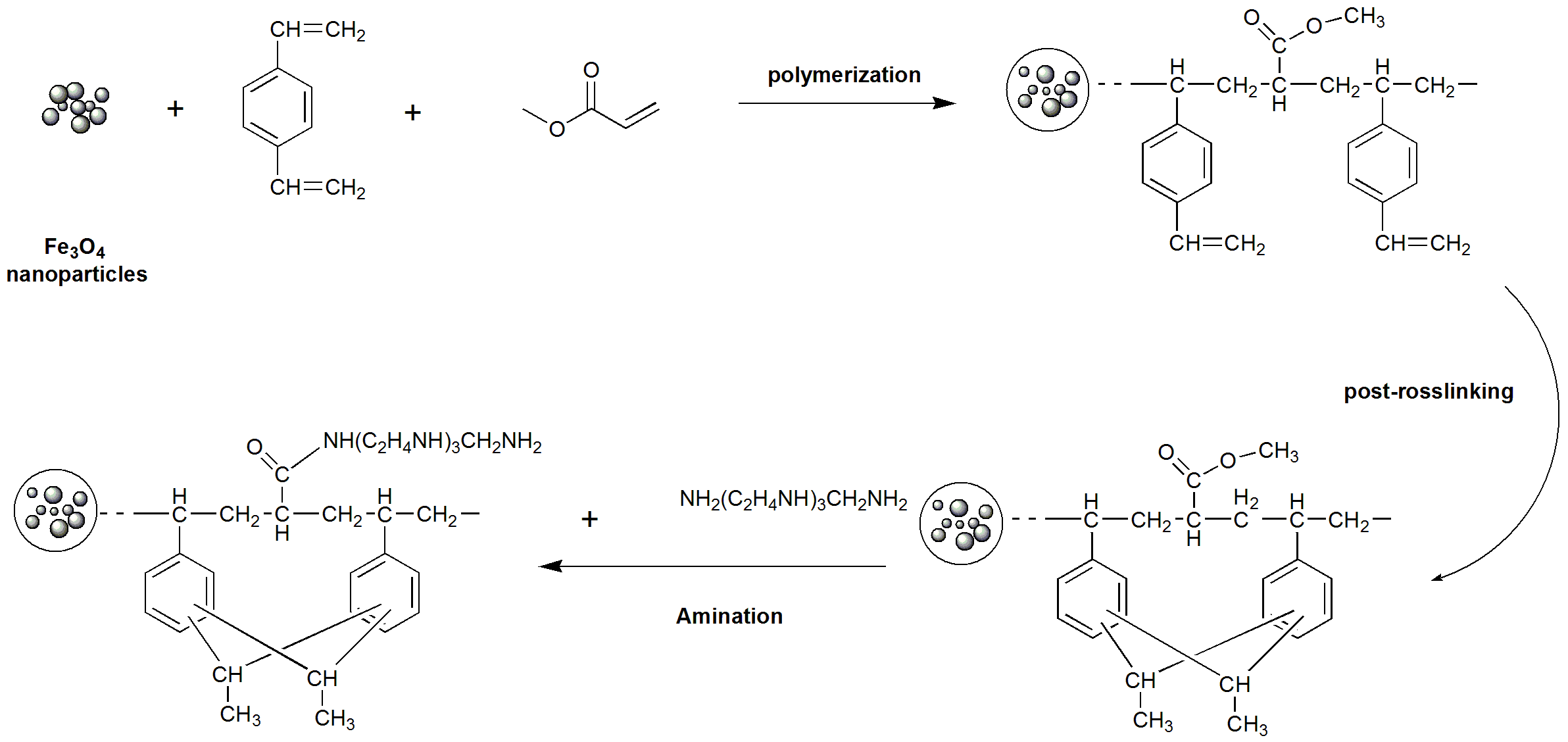


**Figure S1** Preparation procedure of MMAR.


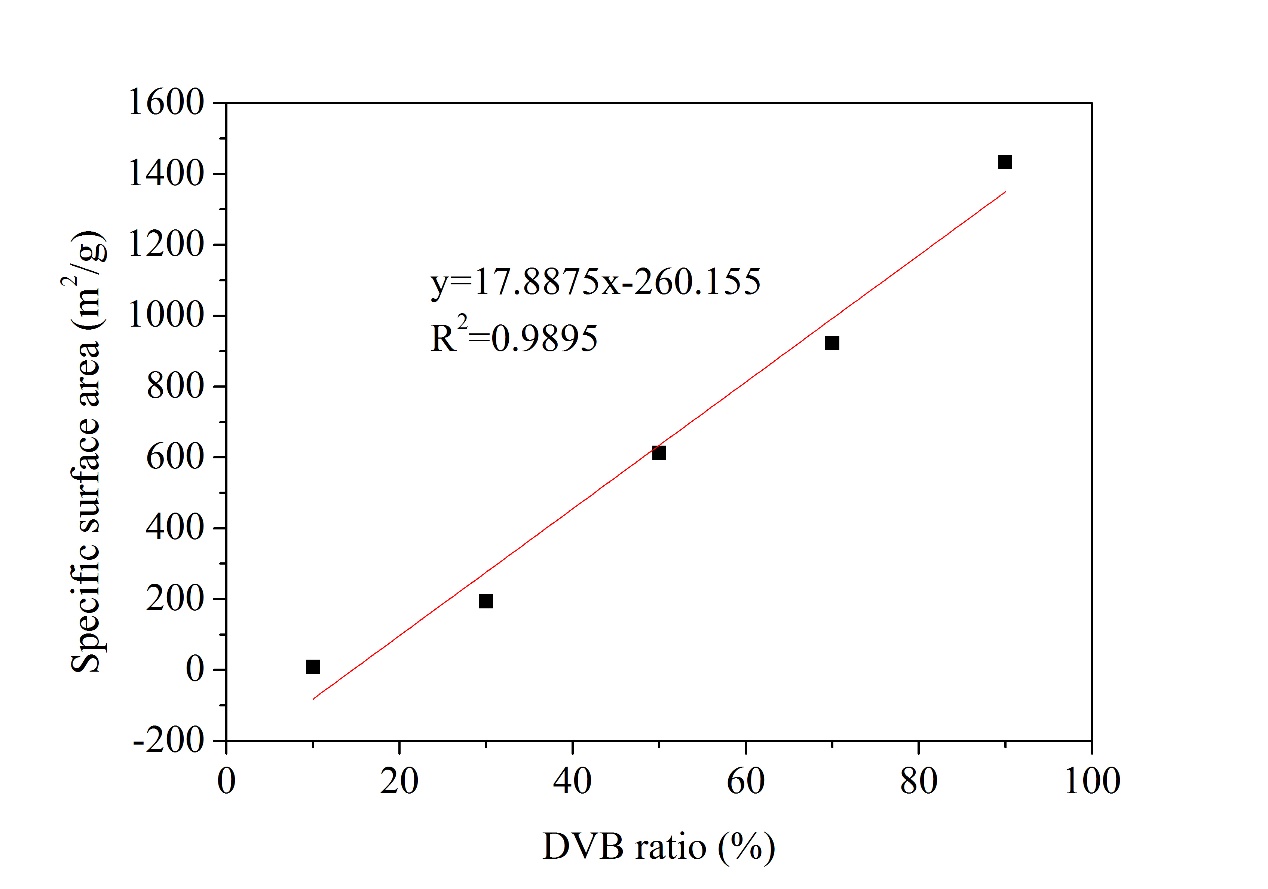


**Figure S2** Linear positive correlation between BET surface areas and DVB ratio.


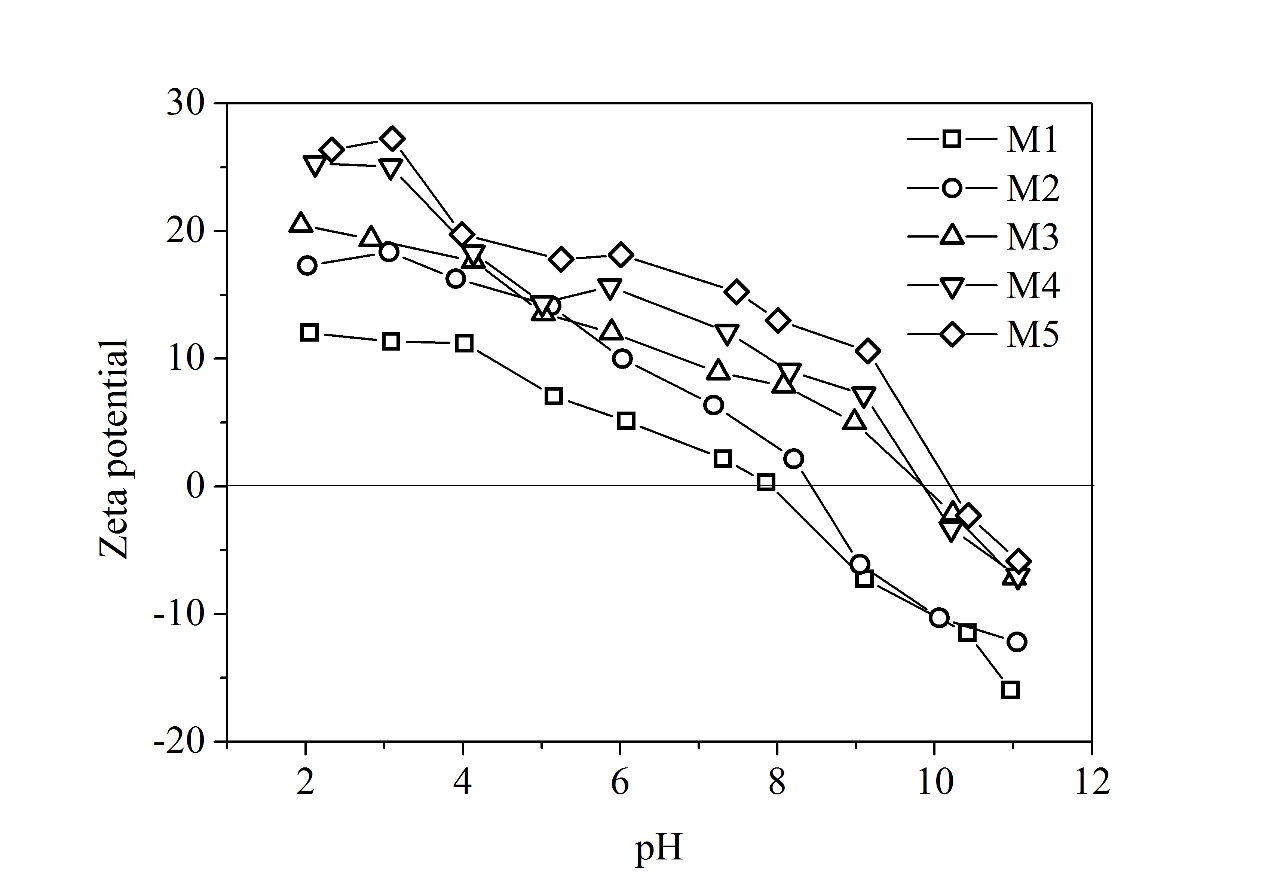


**Figure S3** Zeta potentials of MMARs.





**Figure S4** The speciation diagrams of TC at different pH.

**Table S1** The quality assurance data for the measurement of tetracycline and Cu(II). (μmol/L)

|  | Measurement method | LOD | LOQ | RSD |
| --- | --- | --- | --- | --- |
| TC | UV–visible spectrometry | 0.305 | 1.01 | 0.62 |
| Cu | Atomic absorption spectroscopy | 0.23 | 0.78 | 0.06 |

The quality assurance data for the measurement of TC were obtained according to the Eq. (S1) in the ‘Standard examination methods for drinking water- Water analysis quality control’ (GB/T 5750.3-2006, in Chinese).

LOD=3S_b_/S (S1)

where LOD was the limit of detection. S_b_ represented the standard deviation of multiple measurement of blank samples. S was the slope of calibration curve, representing the sensitivity of the analytical method.

The multi-measurement data were listed in Table S2 and the calibration curve was depicted in Figure S5. Thus, the LOD of TC determination by UV–visible spectrometry was 0.305 μmol/L. The LOQ (limit of quantification), defined as 3.3LOD, was 1.01 μmol/L.

The relative standard deviation (RSD), representing the precision of the analytical method, was 0.62, which was obtained using the Eq. (S2).

RSD=S_b_/Aver. (S2)

where Aver. was the average of the multi-measurement data of blank samples.

Table S2 The multi-measurement data of blank samples.

| multi-measurement data of blank samples | | | | | | | | | | S_b_ | RSD |
| --- | --- | --- | --- | --- | --- | --- | --- | --- | --- | --- | --- |
| 0.003 | 0.003 | 0.001 | 0.003 | 0.003 | 0.001 | 0.002 | 0.004 | 0.005 | 0.002 | 0.001459 | 0.62 |
| 0.003 | 0.004 | 0.004 | 0.000 | 0.002 | 0.004 | 0.001 | 0.000 | 0.002 | 0.000 |  |  |





Figure S5 The calibration curve of TC determined by UV–visible spectrometry.

The Cu(II) concentration was detected according to the ‘Water quality-Determination of copper, zinc, lead and cadmium-Atomic absorption spectrometry’ (GB 7475-87, in Chinese), which clearly reveals the LOQ of 0.05 mg/L or 0.78 μmol/L. The LOD was 0.23 μmol/L. And the RSD, calculated by Eq. (2), was 0.06.
